# Emetine dihydrochloride alleviated radiation‐induced lung injury through inhibiting EMT

**DOI:** 10.1111/jcmm.17959

**Published:** 2023-09-18

**Authors:** Xin Wang, Mo Li, Jizhong Yin, Jiayan Fang, Yimeng Ying, Tianxia Ye, Fangxiao Zhang, Shumei Ma, Hongran Qin, Xiaodong Liu

**Affiliations:** ^1^ Key Laboratory of Radiobiology (Ministry of Health), School of Public Health Jilin University Changchun China; ^2^ Department of Neurology The Third Hospital of Jilin University Changchun China; ^3^ Department of Thyroid Surgery The Second Hospital of Jilin University Changchun China; ^4^ Department of Radiation Medicine, Faculty of Naval Medicine Naval Medical University Shanghai China; ^5^ School of Public Health and Management Wenzhou Medical University Wenzhou China; ^6^ Department of Nuclear Radiation, Shanghai Pulmonary Hospital, School of Medicine Tongji University Shanghai China

**Keywords:** emetine dihydrochloride (EDD), epithelial–mesenchymal transition(EMT), radiation‐induced lung injury(RILI), Smad3

## Abstract

Radiation‐induced lung injury (RILI), divided into early radiation pneumonia (RP) and late radiation‐induced pulmonary fibrosis (RIPF), is a common serious disease after clinical chest radiotherapy or nuclear accident, which seriously threatens the life safety of patients. There has been no effective prevention or treatment strategy till now. Epithelial‐mesenchymal transition (EMT) is a key step in the occurrence and development of RILI. In this study, we demonstrated that emetine dihydrochloride (EDD) alleviated RILI through inhibiting EMT. We found that EDD significantly attenuated EMT‐related markers, reduced Smad3 phosphorylation expression after radiation. Then, for the first time, we observed EDD alleviated lung hyperaemia and reduced collagen deposit induced by irradiation, providing protection against RILI. Finally, it was found that EDD inhibited radiation‐induced EMT in lung tissues. Our study suggested that EDD alleviated RILI through inhibiting EMT by blocking Smad3 signalling pathways. In summary, our results indicated that EDD is a novel potential radioprotector for RILI.

## 1 INTRODUCTION

Radiation‐induced lung injury (RILI) is the most common and fatal complication in clinical thoracic tumour radiotherapy, and nearly 50% of patients suffer from RILI after radiotherapy.^1^ RILI not only causes patients to have severe pulmonary symptoms, but also makes further radiotherapy difficult to carry out, which seriously threatens patients' life safety and affects their quality of life. RILI was mainly divided into radiation pneumonia (RP) and radiation‐induced pulmonary fibrosis (RIPF) after receiving high doses of ionizing radiation (IR). About 30% of these patients will progress from RP to RIPF and eventually die due to pulmonary fibrosis.^2^ In addition, RILI is also a common type of pathological injury in nuclear and radiation emergencies. A large number of seriously injured people died from RILI in the 1986 Chernobyl nuclear power plant accident, and cases of RILI had also been reported at Fukushima nuclear power Plant. Although a lot of researches have been conducted on the prevention and treatment of RILI, no specific and effective drugs have been found so far. Therefore, it is still urgent to find new ways and explore its mechanism to effectively prevent and treat RILI.

The molecular mechanism of RILI has not been fully clarified. It is believed that IR results in significant DNA damage in alveolar epithelial cells and endothelial cells, leading to the recruitment of immune cells and the release of a large number of cytokines. Eventually, the alveolar epithelial‐fibroblast transformation is disrupted, and the fibroblasts synthesize and secrete extracellular matrix (ECM) proteins in the alveolar space.^3^ A great deal of evidence showed that EMT is a key stage in the occurrence and development of RILI. Therefore, it is an effective way to prevent and treat RILI through EMT, but so far, no drugs have been used in clinical practice based on the EMT signalling pathway.

In order to find effective treatment methods for RILI, we used a small molecule natural compound library and luciferase reporter gene (TGF‐β/smad3/smad4, etc.) system to screen 2801 potential drugs, and selected the first 50 in radiation‐induced EMT model for verification, and 20 potentially useful compounds were found, including EDD. EDD is an isoquinoline alkaloid extracted from the ipecac genus plants of Alizarin family. EDD can kill amoebic trophozoites, and also can be used for scorpion stings, showing good biological security. Recent studies have found that EDD has important anti‐tumour effects on bladder cancer^4^ and breast cancer, and its mechanism has been partially elucidated,^5^, ^6^ showing a novel therapy for cancers. And according to the latest research, EDD could be used for developing potent therapeutics with significant activity against SARS‐COV‐2, providing a promising frontline in the fighting against COVID‐19.^7^ However, whether EDD has anti‐radiation properties has never been investigated. In our study, we found EDD could inhibit radiation‐induced EMT of A549 cells as well as ECM production of primary human pulmonary fibroblasts (PHPFs). Mechanistically, EDD alleviated RILI through blocking activation of Smad3. Our findings identified that EDD is a novel inhibitor of the Smad3 signalling, which could be used as a promising lead compound in the development of smad3 inhibitor‐based therapy for RILI.

## 2 MATERIALS AND METHODS

### 2.1 Animals and EDD treatments

The whole protocols were approved by the Ethics Committee of Second Military Medical University, China. Male C57BL/6 mice, 8 weeks old, obtained from the Experimental Animal Centre of the Chinese Academy of Sciences, Shanghai, China, were used for the animal experiment. Mice were fed in daily‐changed individual cages, at 25 ± 1°C with food and water provided for free access. The mice were randomly divided into four groups: group 1, non‐irradiated + PBS control; group 2, irradiation + PBS; group 3, non‐irradiated + EDD; group 4, irradiation + EDD. Either EDD (1 mg/kg) was delivered to the corresponding groups by intraperitoneal injection. Mice were housed three per cage and kept under standard laboratory conditions (22 ± 2°C, 55 ± 10% humidity and 12–12 hr/light–dark cycle), during which sterilized food and water were supplied ad libitum. No animals had to be prematurely killed because of morbidity. EDD (Selleck) was given by intraperitoneal injection 3 days before irradiation at the dose of 1 mg/kg/day and continued for 14 days. Then the mice were killed 4 weeks after irradiation. PBS was administered by intraperitoneal injection at the same time in different groups.

### 2.2 Cell culture and EDD treatments

Human pulmonary epithelial cell line A549(American Type Culture Collection) was cultured in DMEM supplemented with 10% fetal bovine serum (FBS) and 1% solution of penicillin and streptomycin (Invitrogen) in 5% CO_2_ at 37°C in a humidified atmosphere. PHPFs were isolated and established from para‐carcinoma normal lung tissues resected from non‐small cell lung cancer patients who underwent pulmonary lobectomy. Informed written consent had been obtained from each individual who agreed to provide lung tissues for research purposes. To ensure that the lung samples were free of tumour cells, we only selected distal lung tissues away from tumour for experiment. Briefly, distal lung tissues were minced into 3–5 mm^3^ size pieces and digested with collagenase IV (2 mg/mL, Roche, Switzerland) in DMEM medium at 37 °C for 1 h with shaking. Then, the digested tissues were washed with DMEM containing 10% FBS and resuspended in Fibroblast Medium (ScienCell) containing 2% FBS, 1% Fibroblast Growth Supplement (FGS) and 1% solution of penicillin and streptomycin.^8^ PHPFs and A549 cells were pre‐treated with EDD (Selleck) at a concentration of 0.1 μM dissolved in PBS for 48 h after serum starvation, then processed for downstream experiments. For evaluating p‐Smad2/3、N‐cadherin and ZEB1 activation in response to irradiation stimulation, cell lysates were collected 8 h after radiation exposure.

### 2.3 Irradiation

The ^60^Co γ‐rays in the Radiation Centre (Faculty of Naval Medicine, Second Military Medical University) were applied for the radiation exposure. After anaesthetization with 10% chloral hydrate (350 mg/kg), the mice were subjected to whole‐lung irradiation. According to previous studies, we choose 25 Gy with a dose rate of 1 Gy/min whole‐lung irradiation in this study (Figure S1). All radiated animals received a single dose of 25 Gy and were monitored up to 4 weeks post‐irradiation. A549 and PHPFs were radiated with 8 Gy at a dose rate of 1 Gy/min.

### 2.4 Sample collection and initial processing

Six mice from different groups were killed by cervical dislocation after anaesthetization. The left lung was fixed with 4% paraformaldehyde for at least 48 h before histological and immunofluorescence analysis. The rest of the lungs were divided into four parts and kept at −80°C for western blot analysis.

### 2.5 Histopathology

Lung tissues were stained with HE and Masson's trichrome. Five fields per section at ×200 magnifications were randomly selected per mouse and two blinded pathologists independently examined 30 fields per group using Nikon DS‐Fi1‐U2 microscope (Nikon). The mean score of all fields examined was taken as the fibrosis score of each animal.

### 2.6 Western blot

Frozen lung tissue was homogenized in mammalian protein extraction reagent (M‐PER) to prepare a protein sample. Lysates of A549 and PHPFS cells were prepared similarly. The lysates were mixed with 10% SDS‐PAGE then electrophoresis was performed on the same amount based on the concentration. After the electrophoresis, the protein was transferred to a nitrocellulose membrane (Amersham) then blocked with 5% milk for 1 h at room temperature. The proteins were incubated with Snail (1:200; Cell Signalling Tech.), ZEB1(1:200; Cell Signalling Tech.), Twist (1:200; Cell Signalling Tech.), p‐Smad3(1:200; Cell Signalling Tech.), FN (1:200; Cell Signalling Tech.), COL1A1(1:200; Cell Signalling Tech.), N‐cadherin (1:200; Cell Signalling Tech. Danvers, USA), α‐SMA (1:1000; Cell Signalling Tech.), Vimentin (1:1000; Cell Signalling Tech.), F‐actin (1:500; Abcam) and GAPDH (1:1000; Cell Signalling Tech.) at 4°C overnight in a shaker incubator. After washing with TBS‐T, the membranes were incubated with anti‐rabbit or anti‐mouse IgG horseradish peroxidase conjugated antibody (1:5000; Cell Signalling Tech.) for 1 h at room temperature. The protein bands were visualized using enhanced chemiluminescence (ECL) labelling using an ECL Western blot detection system (Thermo Fisher Scientific). The expression of each molecule was normalized to GAPDH expression. The whole uncropped images of the original western blots were shown in Figure S2.

### 2.7 Immunofluorescence staining

Immunofluorescence analysis was performed to measure the expression of E‐cadherin and Vimentin in lung tissues. After deparaffinization, antigen retrieval and incubation with Rodent Block M, the sections were incubated with a mixture of anti‐Vimentin (1:500, Cell Signalling Tech.) and anti‐E‐cadherin (1:500, Cell Signalling Tech.) antibodies at 4°C overnight. After washed with PBS, the sections were incubated with FITC‐conjugated or (Invitrogen) Texas Red‐conjugated anti‐rabbit secondary antibodies (Cell Signalling Tech.) at room temperature for 30 min. Nuclei were counterstained with DAPI, and the sections were analysed using a fluorescence microscope (Nikon Eclipse Ti‐SR).

### 2.8 Cell viability CCK8

Cell viability was qualified with CCK‐8 assay (Cell Counting Kit‐8). More specifically, cells were seeded into 96‐well plates at a density of 5 × 10^3^ per well. 24 h later, cells were treated with EDD and were incubated for another 24 h. After that, CCK‐8 solution was added to each well and the plates were put back to incubator for 2 h. The absorbance of each well was measured at 450 nm with a microplate reader (Thermo Fisher Scientific, Inc.).

### 2.9 Statistical analysis

Data are expressed as the mean ± standard error of the mean (SEM). Comparison between‐group was performed using an one‐way anova. Two‐group comparisons were performed using independent‐samples Student's *t*‐test. *p* < 0.05 was considered significant. All experiments were performed at least three independent times.

## 3 RESULTS

### 3.1 EDD alleviated radiation‐induced fibrotic process of cells

In our study, the non‐toxic dose of EDD was identified by CCK8 assay and was used in the subsequent experiments. The A549 cells (a cell line widely used as human alveolar type II epithelial cells) and BEAS‐2B cells (Human bronchial epithelial cell line BEAS‐2B)were treated with different concentrations of EDD (0.01–10 μM), and cell viabilities were determined by CCK8 (Figure S3). And 0.1 μM of EDD was used in the subsequent experiments.

**FIGURE 1:**
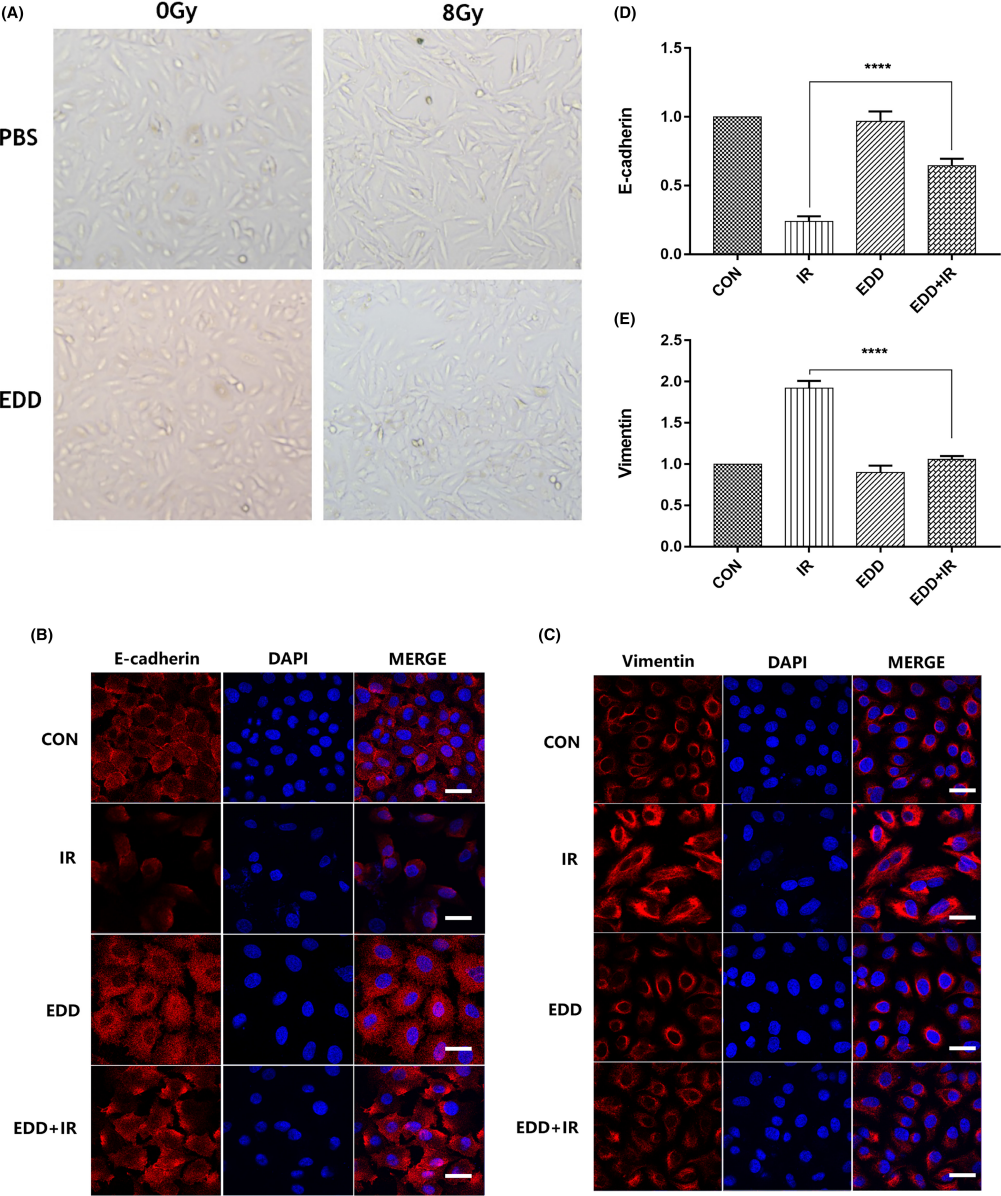
EDD alleviated radiation‐induced fibrotic process of cells. (A) Representative images of phase contrast microscopy images of A549 cells in different groups at 48 h. (B) Representative images of immunofluorescence staining for E‐cadherin (red) in different groups at 48 h post‐irradiation. Scale bars = 10 μm. Original magnification: 630×. (C) Representative images of immunofluorescence staining for Vimentin (red) in different groups at 48 h post‐irradiation. Scale bars = 10 μm. Original magnification: 630×. (D) Quantification analysis of immunofluorescence staining for E‐cadherin. (E) Quantification analysis of immunofluorescence staining for Vimentin. Values are given as mean ± SEM (*n* = 6 for each group), ****p < 0.0001 versus single radiation group.

EMT of type II alveolar epithelial cell (AEC II) is thought to play critical roles in pneumonitis fibrosis. EMT is a pathological process in which epithelial cells transform into mesenchymal cells. After 8Gy γ‐ray irradiation for 48 h, A549 cells exhibited an evident alteration in cell morphology, changing from a classic organized cobblestone epithelial appearance to a disorganized fibroblast‐like phenotype with reduced cell‐to‐cell contact. However, in the presence of EDD, 8Gy irradiated cells maintained an epithelial phenotype (Figure 1A). E‐cadherin is essential for the maintenance of intercellular adhesion and epithelial characteristics, and its loss is one of the markers of EMT. While mesenchymal markers Vimentin and α‐SMA upregulated during EMT. In our study, immunofluorescence staining of E‐cadherin and Vimentin were evaluated for marked loss of epithelial features in A549 cells after irradiation (Figure 1B,C). The results showed that the expression of E‐cadherin was significantly decreased and Vimentin was significantly up‐regulated after irradiation with 8Gy. Whereas EDD (0.1 μM) treated cells maintained the epithelial phenotype, the expression of E‐cadherin was significantly increased and the expression of Vimentin was inhibited (Figure 1D,E). Taken together, these data suggested that EDD has a therapeutic effect on radiation‐induced cell injury.

### 3.2 EDD reduced the expression of EMT markers in A549 cells

**FIGURE 2:**
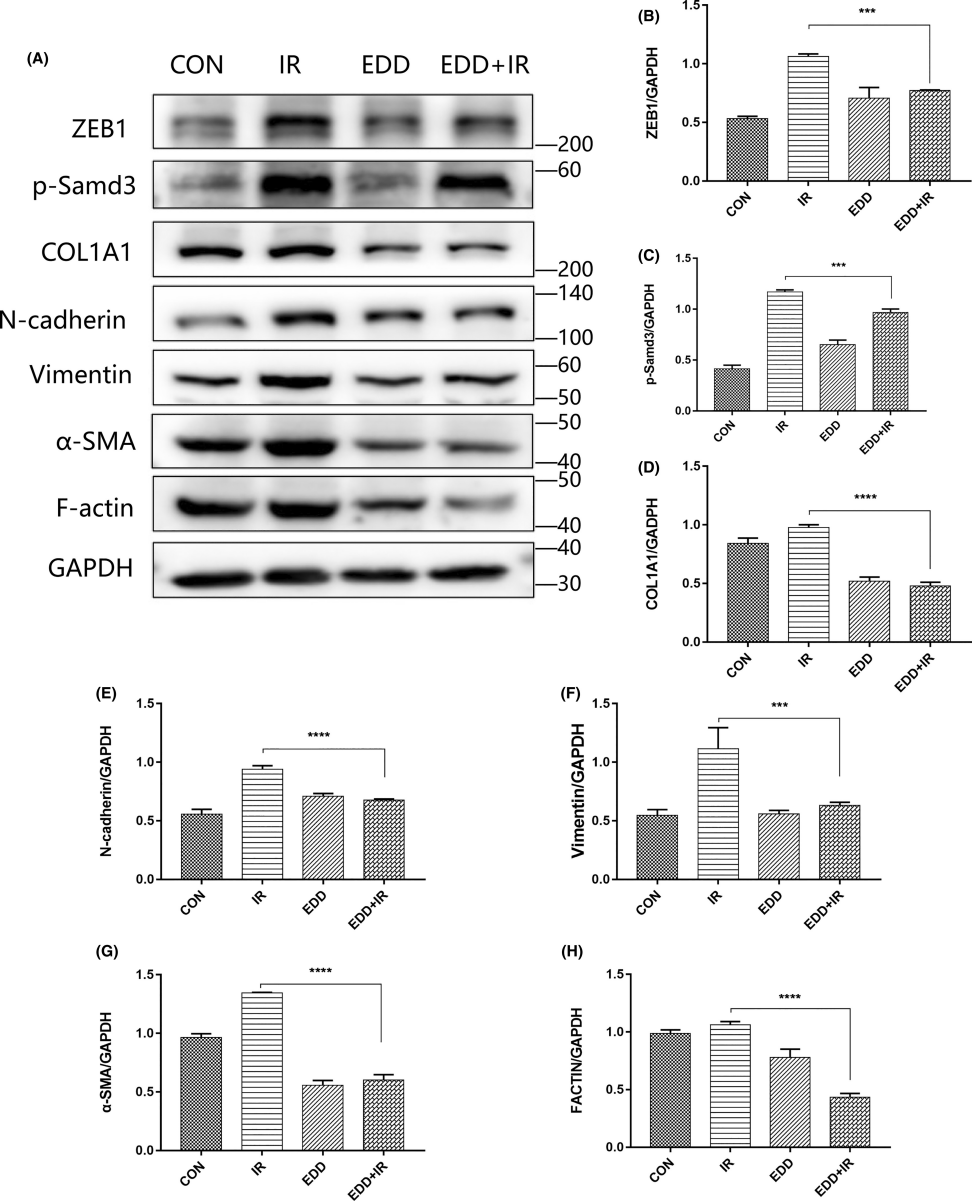
EDD reduced the expression of EMT markers in A549. (A) Western blot analysis of EMT markers in A549 from different groups at 48 h post‐irradiation. Densitometric quantification of ZEB1 (B), p‐Smad3 (C), COL1A1 (D), N‐cadherin (E), Vimentin (F), α‐SMA (G), and F‐Actin (H) protein levels. GAPDH was used as the loading control. The data are presented as the mean ± SEM (*n* = 6 for each group). ***p < 0.001 and ****p < 0.0001 vs. single radiation group.

Radiation‐induced EMT is characterized by the destruction of the intercellular adhesion complex, the disappearance of epithelial cell polarity, and the alteration of skeleton. Therefore, in order to further investigate the mechanism of EDD inhibiting EMT, western blot analysis was performed to compare the related protein expression of γ‐rays treated A549 cells in the presence or absence of EDD (Figure 2A). Previous studies have reported that the occurrence of EMT is associated with Smad3 phosphorylation. By western blot, significantly lower expression of ZEB1 (Figure 2B) and p‐Smad3 (Figure 2C) in EDD‐treated cells were observed than that in the irradiated group. Collagen type I alpha‐1 chain (COL1A1), a key structural protein that plays a binding role in the ECM, also was suppressed after EDD treated (Figure 2D). The results showed that radiation could induce the expression of mesenchymal markers, such as N‐cadherin (Figure 2E), Vimentin (Figure 2F) and α‐SMA (Figure 2G) in A549 cells whereas EDD can significantly blunt radiation‐induced increases. The inhibitory effect of EDD on radiation‐induced EMT was collaborated with reduced expression of F‐actin, an actin stress fibre reflecting EMT‐related cytoskeleton remodelling (Figure 2H). Our data provide novel insight into the radioprotection effect of EDD in radiation‐induced EMT.

### 3.3 EDD suppressed radiation‐induced ECM production and EMT in PHPFs

**FIGURE 3:**
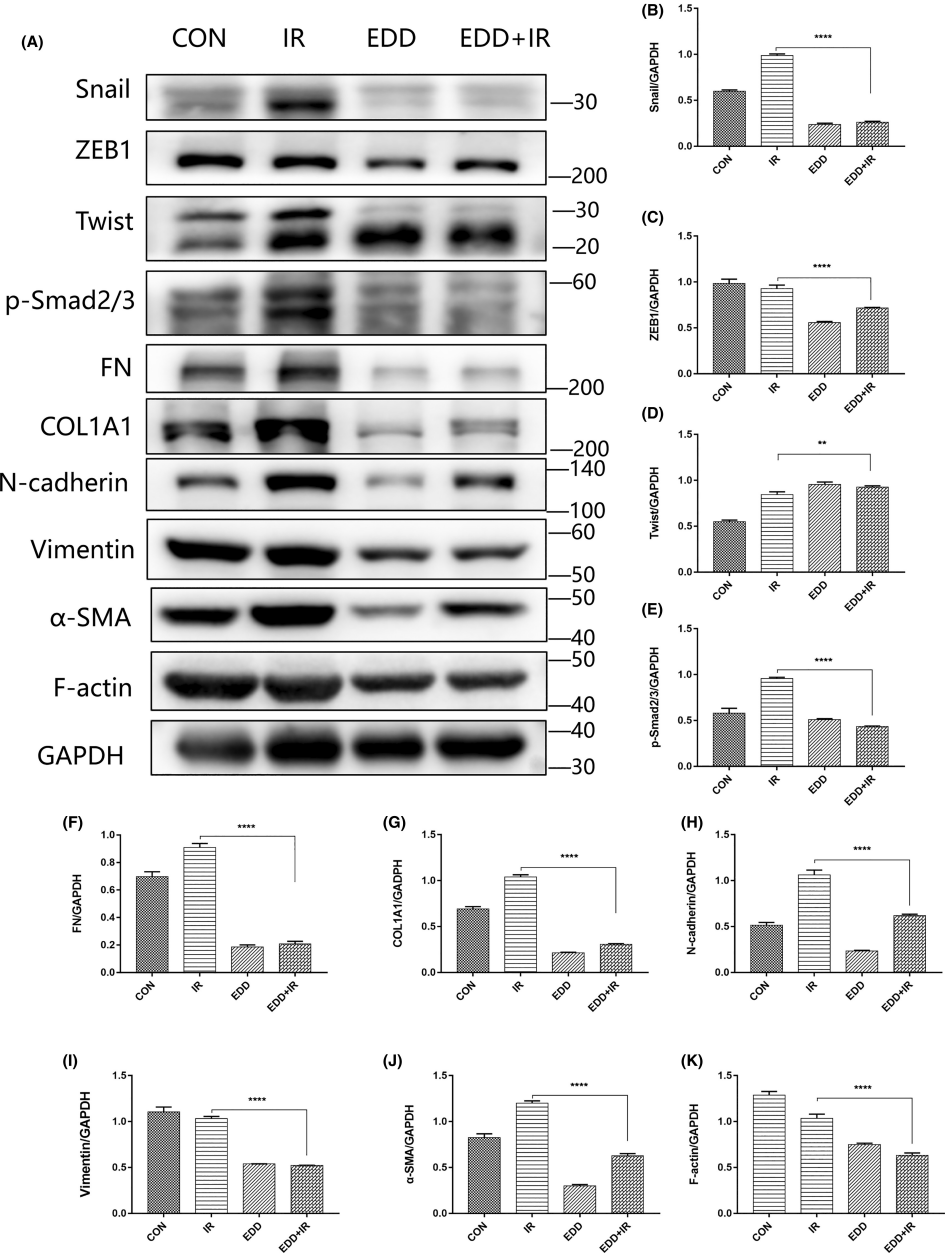
EDD suppressed radiation‐induced ECM production and EMT in PHPFs. (A) Western blot analysis of EMT markers in PHPFs from different groups at 48 h post‐irradiation. Densitometric quantification of Snail (B), ZEB1 (C), Twist (D), p‐Smad2/3 (E), FN (F), COL1A1 (G), N‐cadherin (H), Vimentin (I), α‐SMA (J) and F‐Actin (K) protein levels. GAPDH was used as the loading control. The data are presented as the mean ± SEM (*n* = 6 for each group). **p < 0.01 and ****p < 0.0001 vs. single radiation group.

Accumulation of proliferating fibroblasts and production of excess ECM in fibrotic lesions has been considered the central in fibrogenesis. Therefore, we subsequently examined whether EDD could affect the ECM production and the related mechanisms. PHPFs were isolated from resected lungs and irradiated with 8Gy γ‐rays. Western blot analysis was performed to compare the related proteins expression of γ‐rays treated PHPFs in the presence or absence of EDD (Figure 3A). As expected, after irradiation, EDD‐treated PHPFs exhibited significantly lower expression of transcription factors, including Snail (Figure 3B), ZEB1 (Figure 3C) and Twist (Figure 3D) than single radiated group. Additionally, the expression decline of p‐Smad3 could be detected easily (Figure 3E). Compared with the excess ECM in radiation‐treated PHPFs, EDD significantly decreased the ability of fibroblasts differentiation into myofibroblasts induced by γ‐rays, as demonstrated by reduced expression of ECM proteins, including fibronectin (FN) (Figure 3F), COL1A1 (Figure 3G) and mesenchymal markers, N‐cadherin (Figure 3H), Vimentin (Figure 3I), α‐SMA(Figure 3J). Consistently, F‐actin was reduced by EDD too. Collectively, these data suggest that EDD exert a powerful antifibrotic activity by reducing ECM production in fibroblasts and inhibiting EMT. We demonstrated that the reduction of EMT‐related markers by EDD was associated with the reduced Smad3 phosphorylation and transcription factors.

### 3.4 EDD reduced collagen deposit induced by irradiation in lung tissues

**FIGURE 4:**
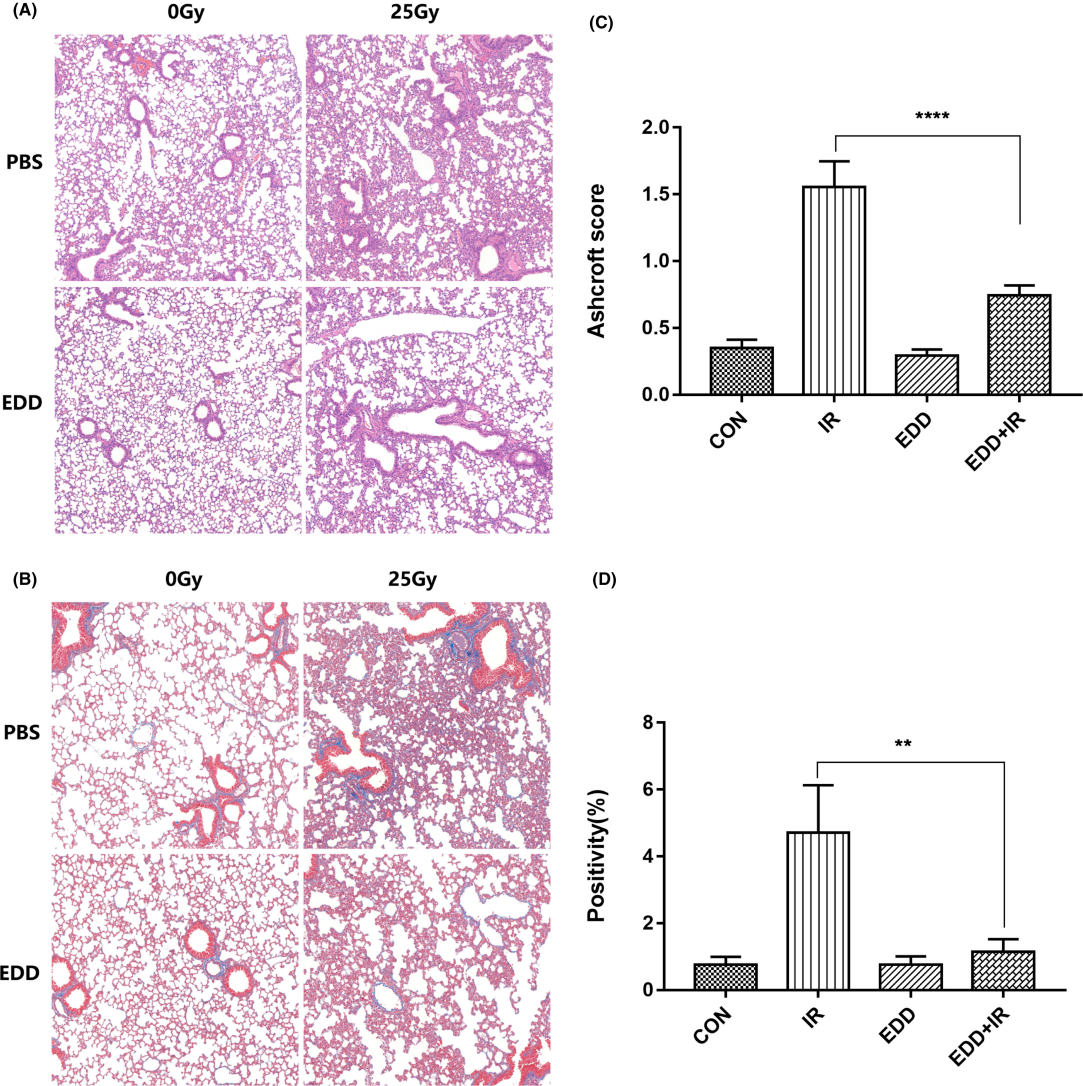
EDD reduced collagen deposit induced by irradiation. Representative images of HE (A) and Masson staining (B) of lung tissue sections at 4 weeks post‐irradiation (*n* = 6). Scale bars = 100 μm. Semiquantitative histological analysis of pulmonary injury and fibrosis. Ashcroft score(C) and quantification of Masson's Trichrome stain for collagen content(D). The data are presented as the mean ± SEM (*n* = 6 for each group). **p < 0.01 and ****p < 0.0001 vs. single radiation group.

To determine the in vivo therapeutic efficacy of EDD on RILI, we established a RILI mice model. The whole‐thorax of C57BL/6 male mice were locally irradiated with 25Gy ^60^Co γ‐rays (Naval Medical University) for a single time under anaesthetized conditions. Lung tissues were taken from the mice at 4 weeks after irradiation and sections were made. HE staining was performed to observe the lung injury under a 100‐fold microscope. We found that radiation induced hyperaemia and oedema of lung tissue, telangiectasis, inflammatory cell infiltration, alveolar collapse, alveolar septum thickening, fibroblast proliferation and even part of the alveolar walls was completely replaced by collagen fibres (Figure 4A). Moreover, collagen deposit in lung tissue was assessed using Masson staining. Interestingly, significant lower levels of collagen deposit were observed in EDD‐treated mice than those in the pure irradiation group (Figure 4B).These data suggested that EDD could reduce the damage of lung structure caused by radiation in mice, inhibit cellulose exudation, and reduce lung collagen deposition as indicated by the Ashcroft score (Figure 4C) and the quantification of Masson staining for collagen content (Figure 4D).

### 3.5 EDD inhibited EMT in radiation‐induced lung injury mice

**FIGURE 5:**
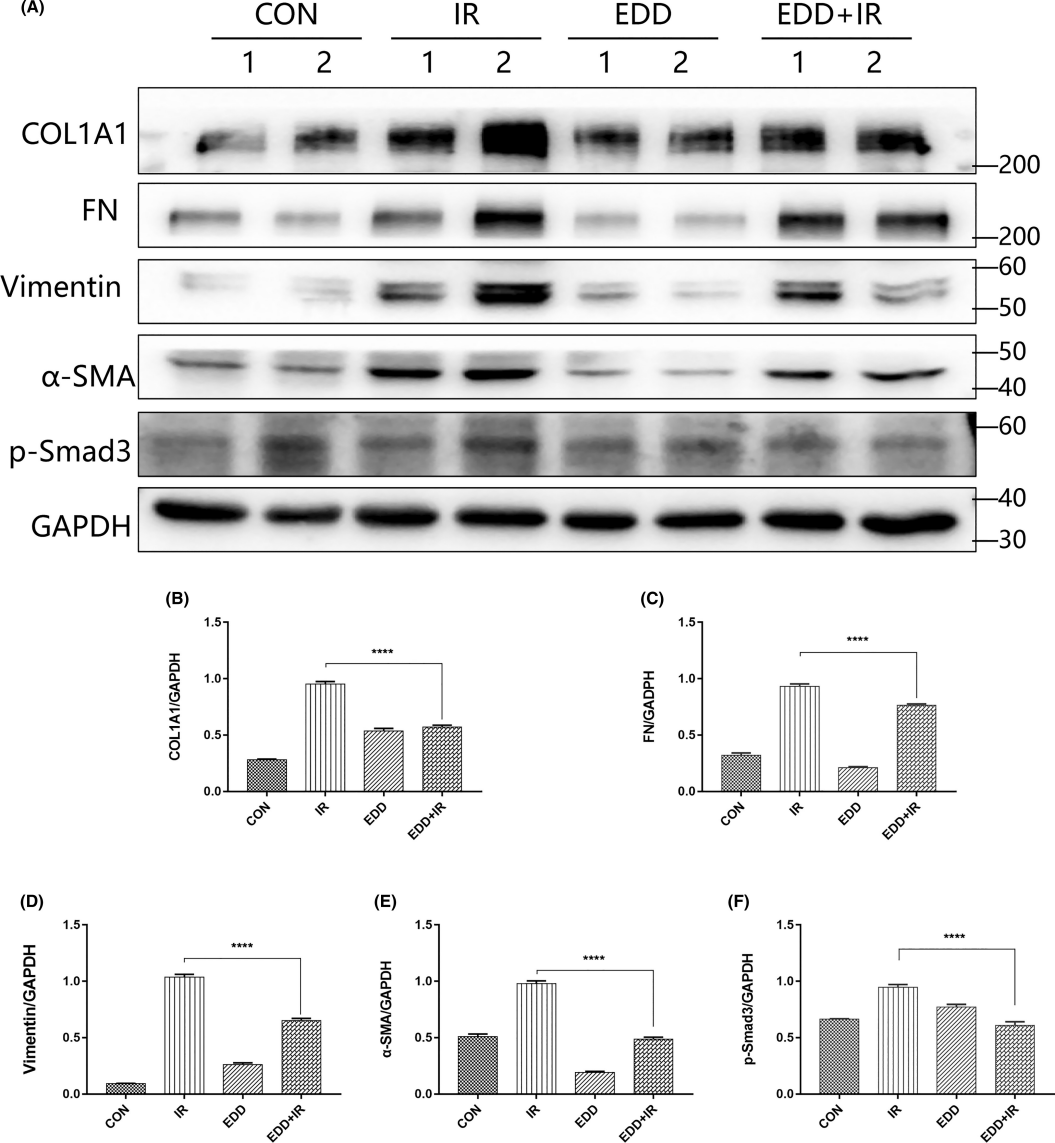
EDD inhibited EMT in radiation‐induced lung injury mice. (A) Western blot analysis of EMT markers in RILI mice from different groups at 4 weeks post‐irradiation. Densitometric quantification of COL1A1 (B), FN (C), Vimentin (D), α‐SMA (E) and p‐Smad3 (F) protein levels. GAPDH was used as the loading control. The data are presented as the mean ± SEM (*n* = 6 for each group). ****p < 0.0001 vs. single radiation group.

To further verify our in vivo findings, we examined the expression of EMT‐related proteins in mice lung tissues. The western blot analysis indicated that EDD inhibited the production of radiation‐induced ECM proteins, COL1A1(Figure 5B) and FN (Figure 5C). In addition, the lung mesenchymal markers, Vimentin (Figure 5D) and α‐SMA (Figure 5E) were substantially decreased after irradiation. At the same time, we found EDD can block p‐Smad3 expression (Figure 5F). Our results demonstrated that EDD played a significant protective role against RILI in mice, providing experimental evidence for its application in vivo.

### 3.6 EDD suppressed TGF‐β/Smad3 pathway in lung tissues

**FIGURE 6:**
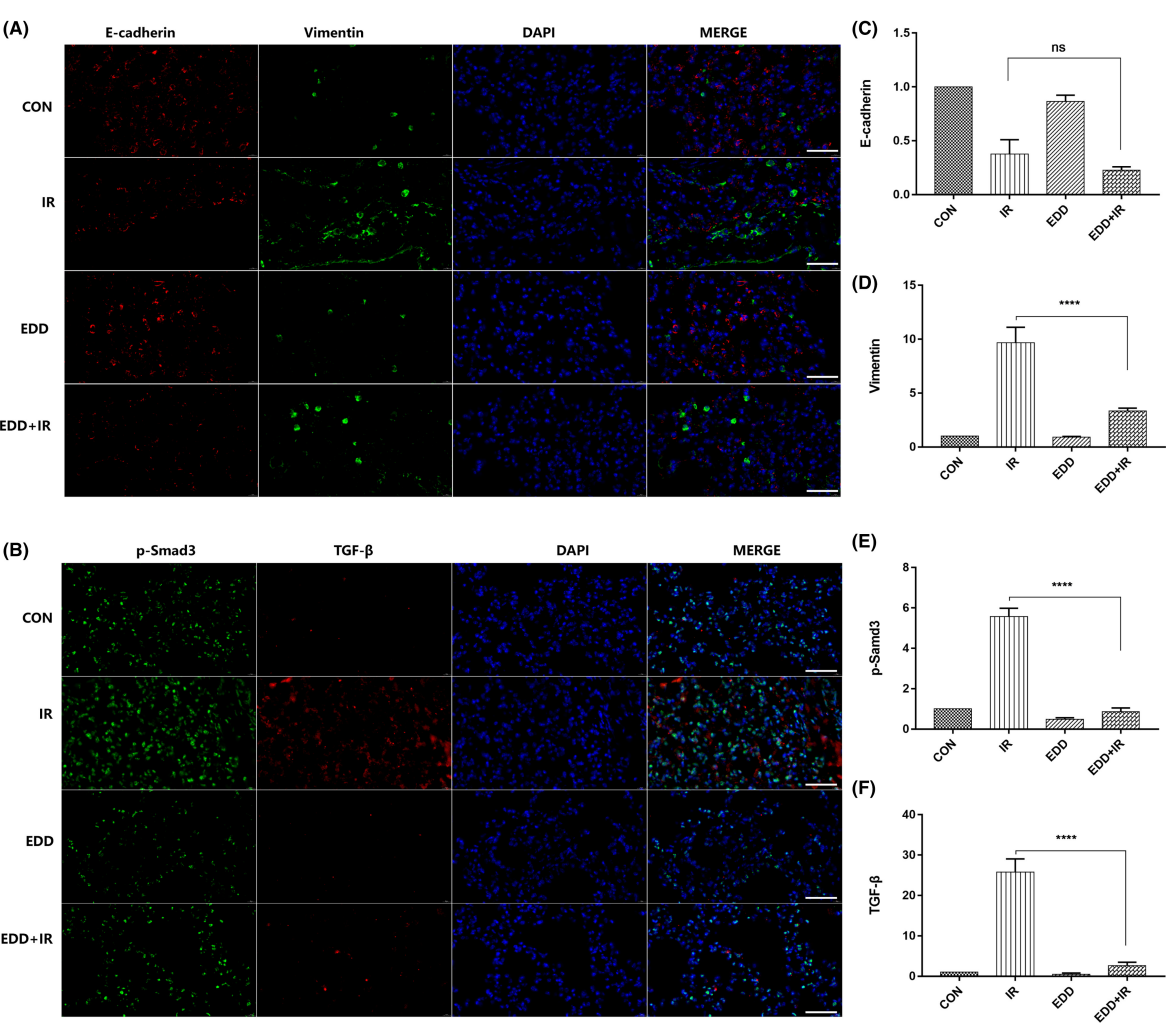
EDD suppressed TGF‐β/Smad3 pathway in lung tissues. A, Representative images of immunofluorescence staining for E‐cadherin (red) and Vimentin (green) in different groups at 4 weeks post‐irradiation. Scale bars = 40 μm. B, Representative images of immunofluorescence staining for p‐Smad3 (green) and TGF‐β (red) in different groups at 4 weeks post‐irradiation. Scale bars = 40 μm. Quantification analysis of immunofluorescence staining for E‐cadherin (C), Vimentin (D), p‐Smad3 (E) and TGF‐β(F). Values are given as mean ± SEM (*n* = 6 for each group), ****p < 0.0001 versus single radiation group.

TGF‐β/Smad signalling pathway plays important roles in EMT. In our study, immunofluorescence staining showed that at 4 weeks after irradiation, the epithelial marker E‐cadherin was progressively down‐regulated and the mesenchymal markers Vimentin were significantly upregulated (Figure 6A). In the EDD treatment group, the upregulation of mesenchymal markers Vimentin and the decrease of E‐cadherin were significantly inhibited (Figure 6C,D). Furthermore, we detected the expression of TGF‐β and p‐Smad3. Consistent with the EMT markers, we found a significant inhibition by EDD on TGF‐β and p‐Smad3 at 4 weeks after irradiation (Figure 6B) as indicated by the quantification for p‐Smad3 (Figure 6E) and TGF‐β (Figure 6F).

## 4 DISCUSSION

RILI is a common complication in patients with thoracic radiotherapy, as well as in patients in nuclear radiation accidents. However no available treatment has yet been found for RILI. The underlying mechanism is even unclear. In our study, we found that EDD effectively mitigated the RILI through inhibiting EMT, which was reported to play critical roles in RILI.^9^ Our results showed that EDD maintained the morphology of epithelial cells, passivated radiation‐induced EMT in A549. We also found that ECM production was decreased and EMT‐related markers were inhibited in EDD‐treated PHPFs after radiation. Irradiation can cause an elevation of transcription factors which play important roles in EMT. Western blot indicated that EDD could reduce radiation induced transcription factors such as Smad3, Snail, ZEB1 and twist. In vivo, EDD inhibited radiation‐induced collagen deposition and showed a good effect to inhibit EMT.

EMT is thought to play critical roles in RILI.^10^ It is characterized with a downregulation of E‐cadherin, and increases of N‐cadherin, Vimentin and α‐SMA. It was reported that TBK1‐mediated Akt‐Erk signal activation can promote the occurrence and development of RILI,^11^ while mesenchymal stem cell therapy,^3^ macrophage colony‐stimulating factor,^12^ Polydatin,^13^ Heat‐killed Salmonella typhimurium,^9^ Grape seed pro‐anthocyanidins^14^ and so on can produce certain prevention and treatment effects by regulating EMT. These findings suggest that it is a good method to treat RILI through EMT. In our study, we found that EDD can maintain epithelial cells morphologically. Additionally, EDD inhibited the upregulation of Vimentin, N‐cadherin and α‐SMA, both in A549 cells and PHPFs after irradiation. COLIA1 and FN are the main components of ECM. ECM production and F‐actin polymerization promote the occurrence of EMT. We found that EDD reduced the production of COLIA1, FN, and F‐actin in PHPFs, indicating that EDD inhibited the EMT process in the late phase of RILI.

Previous studies have revealed that Smad3 signalling pathway plays important roles in EMT, stemness and metastasis of tumours.^15^, ^16^, ^17^ It is reported that renal EMT and tissue fibrosis can be blocked by inhibition of Smad3 activation and nuclear translocation.^18^ For pulmonary fibrosis, EMT can be inhibited both in vivo and in vitro by inhibiting the TGF‐β/Smad pathway.^19^, ^20^ Whereas, collagen synthesis and ECM synthesis were significantly inhibited in Smad3‐deficient cells.^21^ Moreover, Smad3‐deficient mice do not develop pulmonary fibrosis.^22^ Surprisingly, the inhibitory effect of Smad3 on EMT has also been reported in RILI.^23^, ^24^ However, there is little report on the relationship between EDD and Smad3. In this study, we found that EDD significantly down‐regulate p‐Smad3 in A549 and PHPFs. In addition, the same conclusion was found in RILI mice model. These findings showed that EDD might mitigate RILI through Smad3 signalling pathway. Our results suggested that Samd3 might be a novel potential target for EDD.^25^

It is well known that Smad3 regulates EMT by inducing expression of transcription factors. These transcription factors mainly include the Snail family (Snail1, Snail2 (Slug) and Snail3 (Smug)), ZEB (zinc finger E‐box‐binding homeobox) family (ZEB1 and ZEB2), and basic helix–loop–helix (bHLH) Family (Twist1, Twist2 and TCF3). Transcription factors control intercellular adhesion, cell migration, and basement membrane and ECM degradation, and play an evolutionarily conserved central role in the execution of EMTs in a variety of biological environments and organisms. Recently, some studies have pointed out that EMT can be inhibited both in vivo and in vitro by blocking the expression of Snail, Slug, Twist1 and Zeb.^26^, ^27^ Further, in the study of pulmonary fibrosis, it has been found that gallic acid decreased the expression of p‐Smad3 and inhibited the expression of EMT‐related genes, such as N‐cadherin, vimentin, E‐cadherin, Snail1, and Twist1.^28^ In our present study, the expression of Snail, ZEB1 and Twist were also detected besides EMT‐related markers. It was found that EDD could decrease Snail, ZEB1 and Twist induced by irradiation. Therefore, we speculated that EDD could inhibit EMT by down‐regulating the p‐Smad3 expression through blocking the transcription of Snail, ZEB1 and Twist.

In conclusion, our data showed that EDD could alleviate RILI. The mechanism might be related to the inhibition of the EMT process and the blocking of the Smad3 signalling pathway. This study suggested EDD as a safe and effective agent against RILI, indicating great potential in clinical application. It is worth noting that the underlying mechanism of RILI is so complicated that many signal pathways and potential targets are involved in RILI. Thus, we need to do more exploration and discovery to pinpoint other possible signalling pathways and mechanisms of EDD in alleviating lung injury.

## AUTHOR CONTRIBUTIONS

**Xin Wang:** Formal analysis (equal); methodology (lead); writing – original draft (lead). **Mo Li:** Formal analysis (supporting); investigation (equal); software (equal). **Jizhong Yin:** Methodology (equal); software (equal); validation (lead). **Jiayan Fang:** Formal analysis (supporting); investigation (supporting). **Yimeng Ying:** Investigation (supporting); validation (supporting); visualization (supporting). **Tianxia Ye:** Formal analysis (supporting); investigation (supporting); methodology (supporting). **Fangxiao Zhang:** Methodology (supporting); software (supporting); validation (supporting). **Shumei Ma:** Conceptualization (equal); project administration (equal); writing – review and editing (equal). **Hongran Qin:** Funding acquisition (supporting); project administration (equal); writing – review and editing (equal). **Xiaodong Liu:** Conceptualization (equal); funding acquisition (lead); project administration (equal); resources (lead); supervision (equal).

## CONFLICT OF INTEREST STATEMENT

The authors have no conflicts of interest to disclose.

## Supporting information


**Figure S1.** The radiation lung injury model for mice.
**Figure S2.** The original gel blot images of Figure 2, Figure 3 & Figure 5 for checking. A, The original gel blot images for figure2; B, The original gel blot images for Figure3; C, The original gel blot images for figure5.
**Figure S3.** Effects of EDD on cell viability in A549 and BEAS‐2B cells

## Data Availability

The data that support the findings of this study are available from the corresponding author upon reasonable request.
