# Supplementary material for: Emetine dihydrochloride alleviated radiation‐induced lung injury through inhibiting EMT
Source: J Cell Mol Med. 2023 Sep 18;27(23):3839–50. doi: 10.1111/jcmm.17959 (PMC10718159; doi:10.1111/jcmm.17959)
Supplement: Supplementary file 1 — Figure S1. The radiation lung injury model for mice. Figure S2. The original gel blot images of Figure 2, Figure 3 & Figure 5 for checking. A, The original gel blot images for figure2; B, The original gel blot images for Figure3; C, The original gel blot images for figure5. Figure S3. Effects of EDD on cell viability in A549 and BEAS‐2B cells [file JCMM-27-3839-s001.docx]

**Supplementary figures for**

**Emetine dihydrochloride alleviated radiation-induced lung injury through inhibiting EMT**


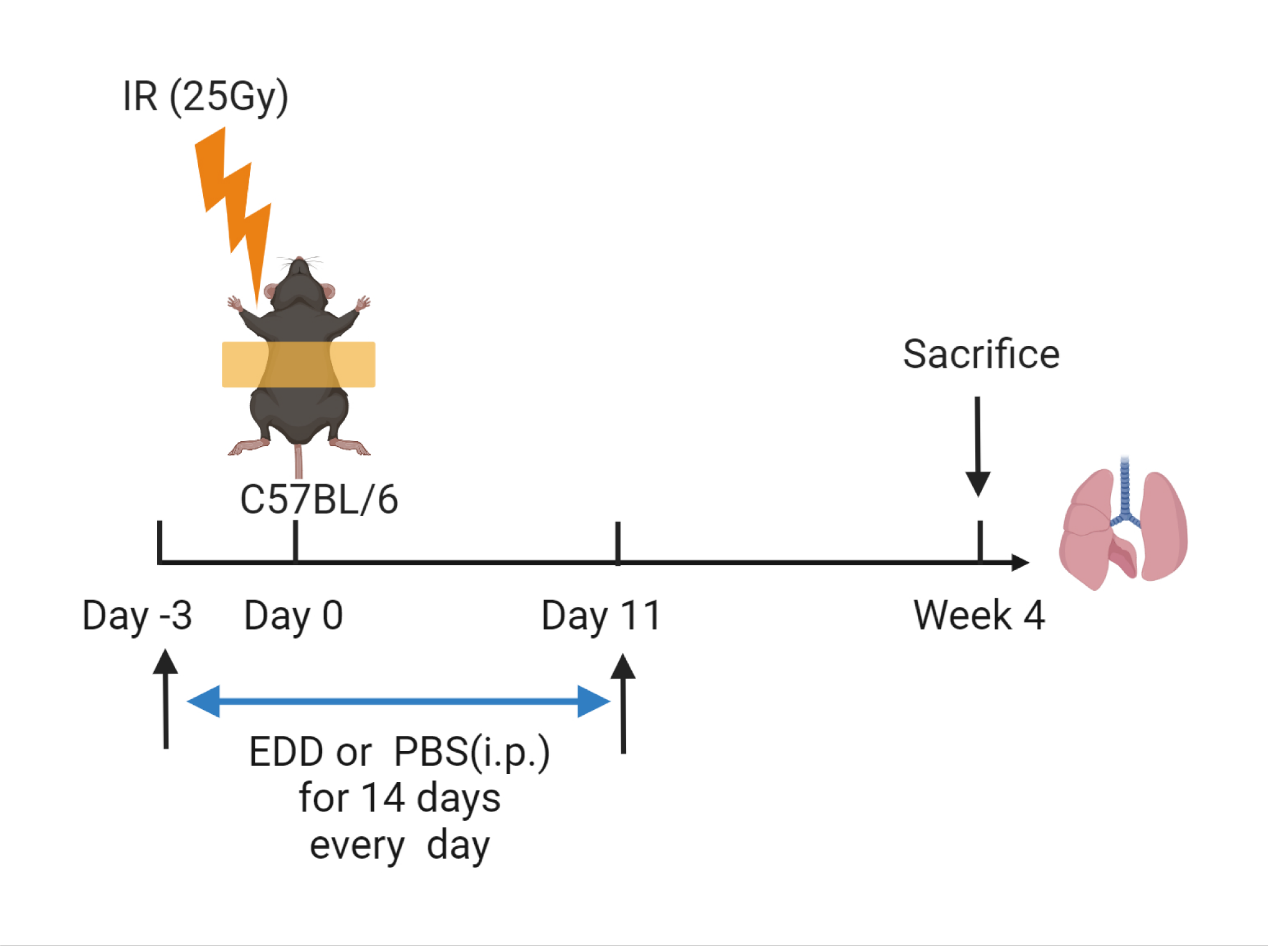
**Supplementary fig. S1.** **The radiation lung injury model for mice.**


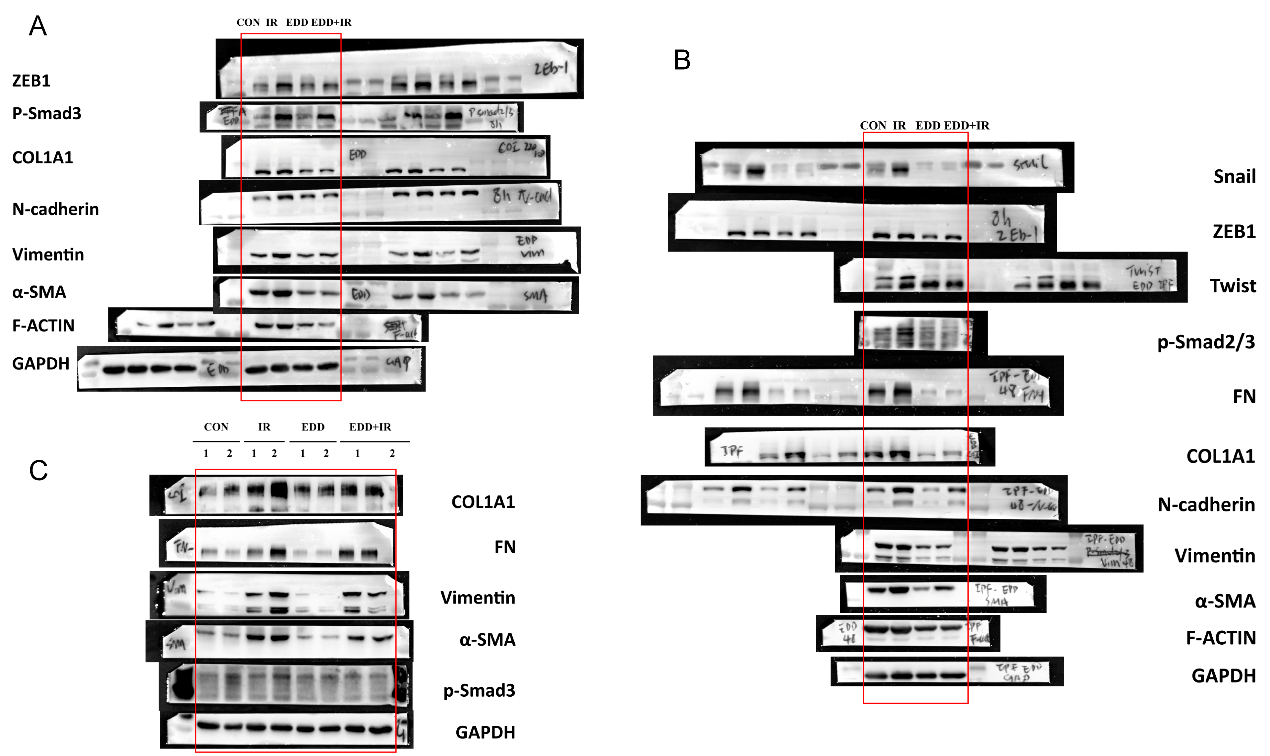


**Supplementary fig. S2.** **The original gel blot images of fig.2, fig.3& fig.5 for cheching. A,** **The original gel blot images for figure2; B,** **The original gel blot images for figure3; C,** **The original gel blot images for figure5.**


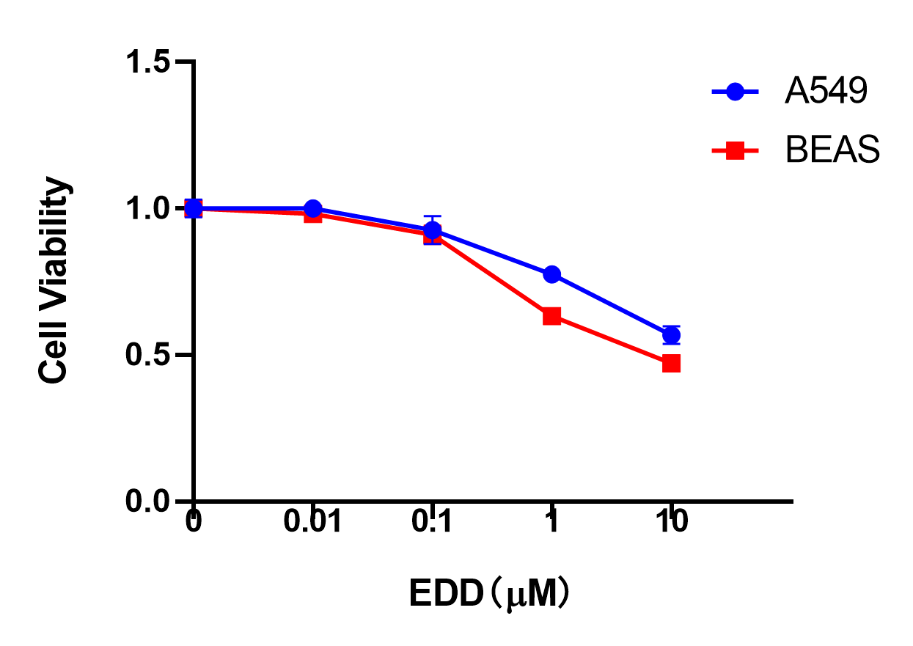


**Supplementary fig. S3. Effects of EDD on cell viability in A549 and BEAS-2B cells**
